# Regional Differences in Disability Incidence among Japanese Adults Aged 75 Years and Older: A 4-Year Prospective Cohort Study

**DOI:** 10.3390/ijerph18136791

**Published:** 2021-06-24

**Authors:** Daisuke Matsumoto, Katsuhiko Takatori

**Affiliations:** 1 Department of Physical Therapy, Faculty of Health Sciences, Kio University, 4-2-2 Umaminaka, Koryo-cho, Kitakatsuragi-gun, Nara 635-0832, Japan; k.takatori@kio.ac.jp; 2 Health Promotion Center, Kio University, 4-2-2 Umaminaka, Koryo-cho, Kitakatsuragi-gun, Nara 635-0832, Japan

**Keywords:** disability, social participation, regional difference

## Abstract

The present prospective study investigated the regional differences and trajectories of new-onset disabilities among older adults in the districts within a city in Japan. We analyzed data from 5050 Japanese residents aged ≥75 years old (men/women: 2512/2538) who completed the Kihon Checklist (a self-reported questionnaire on frailty) and a questionnaire on medical history and social capital in Ikoma city in 2015. The incidence of disability was determined using the new certification of long-term care insurance and was followed-up on 4 years after the primary outcome. A Cox proportional hazards regression model was used to determine the factors related to the risk of incident disability. During the 4-year follow-up period, 567 participants (11.2%) were newly certified to have a disability. The disability incidence rate ranged from 8.1% to 14.6%, depending on the district. After adjustment for the covariates of: older, women, stroke, prefrail, frail, participation in multiple social activities (hazard ratio [HR] = 0.72, 95% confidence interval [CI] = 0.56–0.91), and one of the districts (HR = 1.67, 95% CI = 1.06–2.61) were significantly associated with disability incidence. The findings of this longitudinal study suggest that there could be a regional difference in disability incidence among older adults in Japan. Thus, community-based approaches should be designed to prevent disability in older adults.

## 1. Introduction

By 2050, the worldwide population of individuals aged ≥60 years old is expected to reach 2 billion from the estimated 900 million individuals in 2015. People worldwide are living longer; thus, the pace at which the population is aging is much faster than before [1]. Japan’s population is aging more rapidly than that of any other country [2]. According to government estimates, the percentage of the population over the age of 65 was 26.7% in 2015, and it is expected to exceed 30% in 2025 and reach 39.9% in 2060. In addition, the percentage of those aged ≥75 years is expected to reach 26.9% in 2060. A major challenge of aging in society is the associated high rate of disability [3,4]; hence, public health action on the prevention of disability is urgently needed in almost all countries. Therefore, the identification of high-risk groups and areas where people are prone to disability is important for public health and clinical medicine [5].

Disability is an adverse outcome of frailty [6]. Frailty is a dynamic entity where an individual can transition between the states of being physically active to a functional decline as a result of the loss of dynamic homeostasis, characterized by a high risk of falls, long-term care, and a high mortality rate, as described by the International Conference of Frailty and Sarcopenia Research (ICFSR) guidelines [7]. These guidelines have not been considered from a public health perspective because the focus has been on the management of older people with frailty rather than translating evidence of the condition obtained in studies into clinical practice and healthcare policy, which would make for the successful implementation and improvement of patient care in order to promote healthy aging [7].

In Japan, Makizako et al. reported that physical frailty, even at the pre-frail level, strongly predicted an increased risk of disability [8] and demonstrated the positive effects of community-based disability prevention programs in the older frail population [9]. There are few reports that have emphasized the targeting of preventive strategies in health policies, although some previous studies addressed the risk factors and interventions regarding frailty and disability [6,8,9,10,11,12]. Sato et al. reported that there was a regional disparity in the prevalence of frailty and the number of preventive programs for frailty, with the number of long-term care insurers (as municipalities) being more than twice of those for minimum insurers. Therefore, local governments should promote community-based strategies for the prevention of frailty in communities with older adults at the city level [13]. However, the sphere of daily life for the older population could be smaller, such as a school district instead of an entire city [14]. Previous reports about regional differences in the prevalence of frailty and the incidence of disability at the district level within a city are limited [13].

Thus, we hypothesize that a regional difference in disability incidence might exist even among smaller districts within a city, even after adjustment for confounding factors. Moreover, there might be different trajectories, which can be assessed by a longitudinal study. The present prospective study investigated the regional differences and different trajectories of new-onset disability among older adults in the districts within a city in Japan in order to provide data for evidence-based policy making.

## 2. Materials and Methods

### 2.1. Study Design and Participants

**Figure 1:**
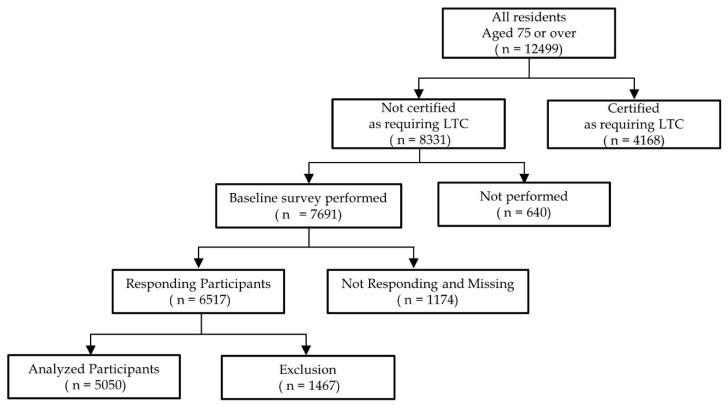
A flow chart of participants in the study cohort. LTC; long-term care.

The study population consisted of community-dwelling elderly people who were ≥75 years old (late-stage elderly people) in Ikoma City, Nara Prefecture, Japan. In 2015, the population of Ikoma City was approximately 120,000, and the population density of the community was approximately 2000 people/km^2^, with an aging rate of approximately 25.0%. As a baseline survey, a postal survey was conducted by the community-based integrated care division of Ikoma City using the Kihon Checklist (KCL), which was sent to 7691 people over the age of 75 in 2015 who were not certified as requiring long-term care. The baseline survey included 6517 older people who responded to the KCL with a response rate of 84.7%, and it excluded 1467 participants who did not provide their responses or had missing data for the outcomes and covariates. A total of 5050 participants (2512 men and 2538 women) were included in the final analysis (Figure 1).

This study was approved by the Ethics Committee of the Kio University (approval number: H28–57). Additionally, the study was conducted in accordance with the provisions of the Declaration of Helsinki and the “Ethical Guidelines for Epidemiological Studies” issued by Ministry of Health, Labour and Welfare, Japan. Personal information was removed from all data by a unique anonymization process performed by the community integrated care section of Ikoma city, which kept researchers blind to all participant personal data.

### 2.2. Measurements of Functional Decline and Frailty Status

The KCL is a questionnaire developed in Japan to identify vulnerable older adults who are at a high risk of becoming dependent. The KCL has been used to predict frailty and disability. It is a self-administered questionnaire that consists of 25 yes/no questions in seven domains, namely the instrumental activities of daily living (IADL) decline, physical decline, malnutrition, oral dysfunction, being homebound, cognitive decline, and depressive mood, as seen in Table S1 [15,16]. Difficulty answering any kind of question was counted as a point on the KCL. A higher score in each domain of the checklist means a higher risk of requiring support or care in a given domain. The assessment of functional decline in each domain is defined as follows: a score of three or more out of the five items in the physical domain (questions number 6–10) indicates a physical decline. A full score of both of the items in the nutritional status domain (questions number 11 and 12) and a score of two or more out of the three items in the oral function domain (questions number 13–15) indicate malnutrition and oral dysfunction, respectively. Homebound is defined by the answer of ‘no’ to question number 16 in the socialization domain (questions number 16 and 17). In addition, a score of one or more out of the three items in the cognitive function domain (questions number 18–20) and a score of two or more out of the five items in the mood domain (questions number 21–25) suggest cognitive decline and depressive mood, respectively [17]. We also defined a score of 10 or more out of the 20 items (questions number 1–20), including the IADL domain (question numbers 1–5), as IADL decline. The total KCL (t-KCL) score is useful for the screening of not only physical frailty but also mental, psychological, and social frailty in older adults as well as for predicting support/care-need certification [18,19]. We considered t-KCL scores of 0–3, 4–7, and 8+ to represent robust, pre-frail, and frail, respectively [20].

### 2.3. Measurements of Other Variables

Other variables were related to individual-level social capital, such as community trust, interactions with neighbours, and types of social participation. Community trust was evaluated on a five-point scale from ‘very important’ to ‘not important’ in response to the question ‘How much do you think trust in neighbours is important in daily life?’ Community trust was assessed as ‘good’ for those who answered ‘very important’ or ‘important’ to the question about trust in neighbours. Interactions with neighbours was rated on a four-point scale from ‘talk with each other and cooperate in terms of life’ to ‘no interaction with neighbours.’ We assessed ‘good’ as interactions for those who answered, ‘cooperate in terms of life’ and ‘talk with each other’. The number of social activities in which subjects participated (i.e., community activities, exercise-based activities, hobby activities, or volunteer activities) ranged from zero (no participation) to four, and subjects participating in two or more types of social activities were considered to be participants of multiple social activities.

### 2.4. Covariates

We collected demographic data including those pertaining to age, sex, living status (alone or otherwise), medical history (hypertension, diabetes mellitus, heart disease, stroke, respiratory disease, and fracture and arthritis), falls, and fear of falls and included these in our analysis as covariates. The history of falls was assessed by having fallen over the past year. Fear of falling was assessed by the presence or absence of anxiety about a future fall.

### 2.5. Outcome Measurements

The Japanese government implemented a mandatory social long-term care insurance (LTCI) system on 1 April 2000. Every Japanese citizen aged ≥65 years is eligible for benefits strictly based on physical and mental disabilities. The computer-aided standardized needs-assessment system categorizes older people into seven levels of needs (Support levels 1–2 and Care levels 1–5), and older people not requiring care were assessed as ‘not certified’. In each municipality, the Care Needs Certification Board, which consists of physicians, public health nurses, and other experts in health and social services, then reviews the results and confirms the individual’s care needs [21]. In this study, we defined new onset of disability as the point at which participants were certified as requiring care according to the LTCI system. During the 4-year post-baseline survey, the participants’ information regarding the new LTCI certifications for those in need of care was obtained by the community integrated care section of the local government.

### 2.6. School District

We chose school districts as the small unit of the community for the following reasons. First, school districts could represent a geographical scale based on whether older adults could travel easily by foot or bicycle and participate in community activities. Second, school districts are useful units that public health practitioners in the local government could use to assess the regional differences in health care indicators within the municipality [14].

### 2.7. Statistical Analysis

The Student’s *t*-test and chi-square test were used to examine the differences in the baseline characteristics between the participants with incidence of disability and those without incidence of disability during the post-baseline assessment at 4 years. To assess the differences among the districts, we aggregated individual-level values for frailty, participation in social activities, and disability incidence in 12 small areas (school districts, A–L). A (lowest) to L (highest) means the ascending order of disability incidence within 4 years. We examined the differences among the districts by using residual analysis after the chi-square test. The presence of more people than expected was considered significant when the adjusted residual values were higher than 1.96, and the presence of fewer people than expected was considered significant when the adjusted residual values were lower than −1.96 [22]. Cox proportional hazards models were used to estimate hazard ratios (HRs) and 95% confidence intervals (CIs) for the risk of incident disability. The first model (Model 1) was adjusted for age, sex, and living alone as covariates by forced entry, while in Model 2, self-reported medical history, the risk of each KCL domain, and frailty status were added to the variables that were included in Model 1. In Model 3, social capital was added to the variables that were included in Model 2. We added the school districts to the variables in Model 3 to generate the final model, Model 4. Models 2, 3, and 4 were developed using stepwise selection. We calculated the cumulative incidence of disability during a 4-year follow-up based on district level with Kaplan–Meier curves. The intergroup difference was estimated by the log-rank test. All data were analyzed using IBM SPSS Statistics for Windows (version 26.0J, IBM Japan Corp., Tokyo, Japan). The level of statistical significance was set at *p* < 0.05.

## 3. Results

After a follow-up period that averaged 3.89 years, 567 people (11.2%) were newly certified as requiring long-term care. Table 1 shows the participants’ baseline characteristics by incident disability during follow-up. Compared with those who remained independent, participants who developed disability were older, more often women, lived alone, and had a higher prevalence of hypertension, heart disease, stroke, fracture, and arthritis. Those with incident disability showed a higher risk in all domains of the KCL than those in the independent group at baseline. The prevalence of frailty in those who developed disability within these 4 years was 29.1%; this value was approximately two-fold higher than that for those who remained independent (13.4%). Those with an incident disability showed lower community trust and participated in fewer types of social activities than did those who were independent.

The prevalence of frailty and the incidence of disability in the school districts are shown in Table 2. There were significant differences in the prevalence of frailty across the districts (district E: 11.8% versus L: 24.2%). With regard to the incidence of disability, there was no significant difference among districts; however, the disability incidence rate was the lowest in district A (8.1%) and the highest in district L (14.6%). The average disability incidence rate was 7.6% (lowest–highest district: 5.3–12.9%) in the robust group, 13.2% (lowest–highest district: 9.8–19.3%) in the pre-frail group, and 21.6% (lowest–highest district: 9.5–31.0%) in the frail group. In addition, the disability incidence rate in the robust group from district J was the highest (12.9%) among all of the groups and districts.

**Figure 2:**
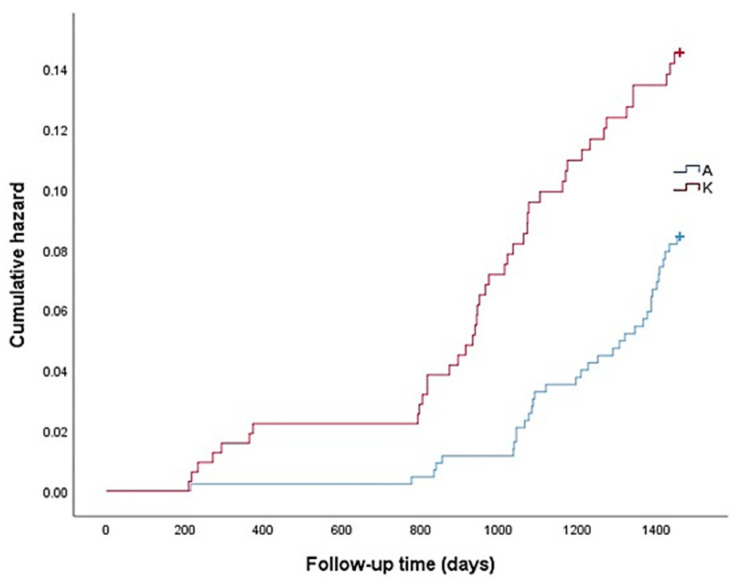
Kaplan–Meier curves for cumulative hazard of incident disability during the 4-year follow-up period in districts A (―) and K (**―**).

Cox proportional hazards analysis was used to determine the factors related to the risk of incident disability (Table 3). In Model 2, stroke and frailty status were selected as significant variables. In Model 3, participation in multiple social activities was selected as a variable related to social capital. In the final model, we made adjustments for age, sex, living alone, medical history, frailty status, participation in social activities, and district and we found that older age, the female sex (HR 1.28, 95% CI 1.07–1.55), stroke (HR 1.84, 95% CI 1.09–3.12), pre-frail status (HR 1.45, 95% CI 1.15–1.82), frail status (HR 1.91, 95% CI 1.33–2.74), and district K (HR 1.67, 95% CI 1.06–2.61) were related to an increased risk of incident disability. While district L showed the highest disability incidence according to cross-sectional data, it was not selected as a significant high-risk area. Alternatively, participation in multiple social activities was related to a decreased risk of incident disability. Living alone remained insignificantly associated with the incidence of disability. Kaplan–Meier curves (Figure 2) on the incidence rate of disability illustrated significantly different trajectories between districts A and K (*p* = 0.011), with the risk of incident disability being higher in district K than in district A (A: 8.1% vs. K: 13.5%).

## 4. Discussion

In this study, we analyzed the differences in disability incidence among community-dwelling older adults in districts of Ikoma City over a 4-year follow-up period. The disability incidence rate was 11.2%, which was slightly higher than those reported in previous cohort studies [8,18]; this can be ascribed to our older study population and the longer follow-up period. Taniguchi et al. reported that the group with a higher-level of early-onset decreasing trajectory of functional capacity increased dramatically over age 75 years of age, and the estimated medical and LTCI costs exceeded those in the lower-level group [23]. Upon comparison of the groups, participants who developed disability showed worse results in terms of almost all of the variables, such as age, sex, medical history, frailty status, and social capital. These results are similar to those of previous studies [3,5]. We found that the disability incidence rate differed from 8.1% (district A) to 14.6% (district L) within the city. One of the reasons was that the prevalence of frailty in district L (24.2%) was higher than reported in a previous systematic review [10].

According to the Cox proportional hazards analysis, old age, the female sex, stroke, pre-frail status, frail status, and district K were related to an increased risk of disability incidence. A recent systematic review and meta-analysis demonstrated pooled evidence that age and stroke were significantly associated with frailty among community-dwelling older people [11]. In addition, stroke was significantly associated with the onset and recurrence of disability [24]. Moreover, studies have shown that older frail people were more likely to develop or progress to worsened disabilities. Prefrailty was also associated with incident or worsening disability risks to a lesser degree in the pooled analyses [6]. Accordingly, our results support the findings from previous reports. Those with an incident disability showed a higher risk in all domains of the KCL than those in the independent group at baseline. Satake et al. reported that both the physical and nutrition domains in the KCL could significantly predict incident dependency and all-cause mortality [17]. However, in our study, these KCL domains could not be selected as significant variables because a patient’s frail status was more strongly associated with incident disability than with each of those domains. In addition, participation in multiple social activities was related to a decreased risk of incident disability. Our results confirmed the findings of previous studies; when participation in no social activity was compared with that in multiple social activities, the latter showed a more effective association with preventive disability [25]. Living alone was not significantly associated with the incidence of disability; however, in a previous systematic review and meta-analysis, it was found that living alone was significantly associated with the probability of developing frailty, but only among men [12]. Conversely, living alone was shown to be associated with decreasing frailty in another study [26], although there was a substantial association between living alone and incidents of disability, and it is possible that adjustment for the other variables might make this association weaker.

Further, we found that the incident rate of disability illustrated significantly different trajectories of functional capacity between intra-city districts among the participants. Previous studies have observed regional differences in the prevalence of frailty and disability incidence among regions and countries [27] and in urban–rural areas among older people in China [28]. Generally, public health practitioners assess each indicator across districts by population groups or geographical areas and make policy choices to prioritize health inequalities within the city [29]. Our notable finding was that there was a regional difference in disability incidence rates across the districts within Ikoma City among older adults in Japan, according to our longitudinal data, even after adjustments for covariates, including frail status and social capital. Furthermore, our cross-sectional data showed that the high-risk disability incidence area (district K) was different from the high prevalence disability incidence area (district L). This study might contribute a solution toward evidence-based health policy.

A major strength of this study is that it contained a large population-based sample of more than 5000 older adults aged ≥75 years old in Japan. However, this study has the following limitations: First, we could not consider confounding factors such as the participants’ exercise habits, social activities [30], and community-based programs conducted by long-term care insurers [13,31] in the period of investigation. Second, we had no information about socioeconomic status (education and income) and geographical data (access and walkability). These unmodifiable factors might explain the causes of regional differences in disability incidence among districts [32]. Future studies should consider examining the causes of regional differences using socioeconomic and geographical data to enable the development of preventive strategies for disability at the level of the local government. Third, medical history (stroke, heart disease, and other medical conditions) was self-reported in the questionnaire, and these data do not guarantee the health literacy of all participants. Finally, we collected and analyzed data and obtained results from a typical but still only one city in Japan; therefore, we should consider these issues carefully while interpreting the results.

## 5. Conclusions

Our results of this prospective cohort study show that there could be a regional difference in disability incidence among districts within a city. Our findings may contribute to the development of more effective community-based assessments and approaches for the prevention of disability in the older population in Japan.

## Figures and Tables

**Table 1:** Baseline characteristics of participants by incidence of disability over the 4 years after baseline assessment.

Variable	Total		Independent		Incident Disability		*p*-Value
	(n = 5050)		(n = 4483)		(n = 567)		
Age, years							<0.001
75–79, n (%)	3002	(59.4)	2777	(61.9)	255	(39.7)	
80–84, n (%)	1475	(29.2)	1273	(28.4)	202	(35.6)	
85–89, n (%)	489	(9.7)	378	(8.4)	111	(19.6)	
90+, n (%)	84	(1.7)	55	(1.2)	29	(5.1)	
Female, n (%)	2538	(50.3)	2197	(49.0)	341	(60.1)	<0.001
Living alone, n (%)	765	(15.1)	659	(14.7)	106	(18.7)	0.008
Medical history							
Hypertension, n (%)	2267	(44.9)	1995	(44.5)	272	(48.0)	0.064
Diabetes mellitus, n (%)	563	(11.1)	492	(11.0)	71	(12.5)	0.151
Heart Disease, n (%)	566	(11.2)	489	(10.9)	78	(13.8)	0.027
Stroke, n (%)	76	(1.5)	61	(1.4)	15	(2.6)	0.02
Respiratory Disease, n (%)	223	(4.4)	192	(4.3)	31	(5.5)	0.12
Fracture and Arthritis, n (%)	689	(13.6)	578	(12.9)	111	(19.6)	<0.001
KCL assessment ^†^							
IADL decline, n (%)	210	(4.2)	154	(3.4)	56	(9.9)	<0.001
Physical decline, n (%)	812	(16.1)	640	(14.3)	172	(30.3)	<0.001
Malnutrition, n (%)	103	(2.0)	82	(1.8)	21	(3.7)	0.004
Oral dysfunction, n (%)	885	(17.5)	751	(16.8)	134	(23.6)	<0.001
Homebound, n (%)	274	(5.4)	212	(4.7)	62	(10.9)	<0.001
Cognitive decline, n (%)	1515	(30.0)	1307	(29.2)	208	(36.7)	<0.001
Depressive mood, n (%)	1357	(26.9)	950	(21.2)	189	(33.3)	<0.001
History of fall (past a year), n (%)	851	(16.9)	713	(15.9)	138	(24.3)	<0.001
Fear of falling, n (%)	2091	(41.4)	1769	(39.5)	322	(56.8)	<0.001
Frailty ^‡^							
Robust, n (%)	2901	(57.4)	2681	(59.8)	220	(38.8)	<0.001
Pre-frailty, n (%)	1384	(27.4)	1202	(26.8)	182	(32.1)	
Frailty, n (%)	765	(15.1)	600	(13.4)	165	(29.1)	
Social capital							
Community trust, n (%)	4332	(85.8)	3864	(86.2)	468	(82.5)	0.013
Interaction with neighbors, n (%)	3571	(70.7)	3184	(71.0)	387	(68.3)	0.095
Social participation, n (%)	2632	(52.1)	2387	(53.2)	245	(43.2)	<0.001
multiple, n (%)	1129	(22.4)	1048	(23.4)	81	(14.3)	<0.001

KCL: Kihon Checklist; IADL: Instrumental Activity of Daily Living. ^†^ Based on KCL sub-score of each domain, ^‡^ Frailty: Out of total KCL score, 0–3 for robust, 4–7 for pre-frailty, and ≥8 for frailty.

**Table 2:** Prevalence of Frailty and disability incidence among the districts.

	Total	A	B	C	D	E	F	G	H	I	J	K	L
Participants, n (%)	5050 (100.0)	433 (8.6)	290 (5.7)	636 (12.6)	582 (11.5)	549 (10.9)	535 (10.6)	330 (6.5)	368 (7.3)	483 (9.6)	286 (5.7)	318 (6.3)	240 (4.8)
Frailty ^†^													
Robust at baseline, n (%)	2901 (57.4)	265 (61.2)	166 (57.2)	343 (53.9)	332 (57.0)	345 (62.8)	311 (58.1)	189 (57.3)	222 (60.3)	266 (55.1)	170 (59.4)	174 (54.7)	118 (49.2)
Prefrail at baseline, n (%)	1384 (27.4)	115 (26.6)	82 (28.3)	193 (30.3)	163 (28.0)	139 (25.3)	140 (26.2)	83 (25.2)	90 (24.5)	147 (30.4)	74 (25.9)	94 (29.6)	64 (26.7)
Frail at baseline, n (%)	765 (15.1)	53 (12.2)	42 (14.5)	100 (15.7)	87 (14.9)	65 * (11.8)	84 (15.7)	58 (17.6)	56 (15.2)	70 (14.5)	42 (14.7)	50 (15.7)	58 ** (24.2)
Adjusted residual	*p* = 0.009	−1.80	−0.30	0.40	−0.10	−2.30	0.40	1.30	0.00	−0.40	−0.20	0.30	4.00
Disability													
follow-up years	3.89	3.94	3.91	3.92	3.91	3.89	3.91	3.87	3.87	3.89	3.88	3.81	3.84
Disability within 4 years, n (%)	567 (11.2)	35 * (8.1)	25 (8.6)	61 (9.6)	58 (10.0)	61 (11.1)	62 (11.6)	39 (11.8)	47 (12.8)	63 (13.0)	38 (13.3)	43 (13.5)	35 (14.6)
Adjusted residual	*p* = 0.102	−2.17	−1.45	−1.40	−1.03	−0.09	0.28	0.35	0.97	1.33	1.14	1.34	1.69
Disability from Robust within 4 years, n (%)	220 (7.6)	14 (5.3)	10 (6.0)	18 (5.3)	26 (7.8)	29 (8.4)	22 (7.1)	13 (6.9)	21 (9.5)	23 (8.7)	22 ** (12.9)	15 (8.6)	7 (5.9)
Adjusted residual	*p* = 0.178	−1.48	−0.78	−1.74	0.18	0.62	−0.36	−0.38	1.10	0.69	2.72	0.53	−0.69
Disability from Prefrail within 4 years, n (%)	182 (13.2)	13 (11.3)	11 (13.4)	22 (11.4)	16 (9.8)	18 (12.9)	20 (14.3)	16 (19.3)	11 (12.2)	22 (15.0)	9 (12.2)	14 (14.9)	10 (15.6)
Adjusted residual	*p* = 0.839	−0.61	0.07	−0.78	−1.34	−0.07	0.42	1.70	−0.27	0.69	−0.26	0.52	0.60
Disability from frail within 4 years, n (%)	165 (21.6)	8 (15.1)	4 (9.5)	21 (21.0)	16 (18.4)	14 (21.5)	20 (23.8)	10 (17.2)	15 (26.8)	18 (25.7)	7 (16.7)	14 (28.0)	18 (31.0)
Adjusted residual	*p* = 0.303	−1.19	−1.95	−0.15	−0.77	−0.01	0.53	−0.83	0.99	0.89	−0.80	1.14	1.82
Disability, 1000 person-year	28.8	20.5	22.1	24.5	25.5	28.6	29.7	30.5	33	33.6	34.3	35.5	38

^†^ Frailty: Out of total KCL score, 0–3 for robust, 4–7 for pre-frailty, and ≥8 for frailty. * *p* < 0.05; ** *p* < 0.01.

**Table 3:** Cox proportional hazards analysis for incident disability.

Variable	Model 1		Model 2		Model 3		Model 4	
	HR (95% CI)	*p*-Value	HR (95% CI)	*p*-Value	HR (95% CI)	*p*-Value	HR (95% CI)	*p*-Value
Age, years								
75–79	ref.		ref.		ref.		ref.	
80–84	1.89 (1.56–2.28)	<0.001	1.72 (1.42–2.09)	<0.001	1.70 (1.41–2.06)	<0.001	1.72 (1.42–2.08)	<0.001
85–89	3.31 (2.63–4.15)	<0.001	2.67 (2.11–3.38)	<0.001	2.64 (2.08–3.34)	<0.001	2.67 (2.10–3.38)	<0.001
90+	5.59 (3.79–8.23)	<0.001	3.81 (2.53–5.72)	<0.001	3.71 (2.46–5.57)	<0.001	3.74 (2.48–5.64)	<0.001
Female	1.41 (1.18–1.67)	<0.001	1.30 (1.08–1.56)	0.005	1.29 (1.07–1.56)	0.007	1.28 (1.07–1.55)	0.005
Living alone	1.11 (0.89–1.38)	0.345	1.13 (0.91–1.41)	0.261	1.15 (0.92–1.43)	0.22	1.16 (0.93–1.44)	0.198
Medical history								
Stroke			1.80 (1.07–3.05)	0.028	1.84 (1.09–3.11)	0.023	1.84 (1.09–3.12)	0.023
Frailty ^†^								
Robust			ref.		ref.		ref.	
Pre-frailty			1.47 (1.17–1.85)	0.001	1.44 (1.14–1.81)	0.002	1.45 (1.15–1.82)	0.002
Frailty			1.99 (1.39–2.84)	<0.001	1.89 (1.32–2.71)	<0.001	1.91 (1.33–2.74)	<0.001
Social capital								
Social participation					0.71 (0.56–0.90)	0.006	0.72 (0.56–0.91)	0.007
(multiple)								
District								
A							ref.	
K							1.67 (1.06–2.61)	0.026

† Frailty: Out of total KCL score, 0–3 for robust, 4–7 for pre-frailty, and ≥8 for frailty. Model 1: age, sex and living alone. Model 2: Model 1 + hypertension, diabetes mellitus, heart disease, stroke, respiratory disease, fracture, arthritis, seven domains of KCL and frail status. Model 3: Model 2 + community trust, interaction with neighbors, and social participation (multiple). Model 4: Model 3 + 12 districts.

## Data Availability

Data sharing not applicable because they contain information that could compromise the privacy of the participants.

## Supplementary Materials

The following are available online at https://www.mdpi.com/article/10.3390/ijerph18136791/s1, Table S1: The Kihon Checklist in English.
