# Supplementary material for: Regional Differences in Disability Incidence among Japanese Adults Aged 75 Years and Older: A 4-Year Prospective Cohort Study
Source: Int J Environ Res Public Health. 2021 Jun 24;18(13):6791. doi: 10.3390/ijerph18136791 (PMC8297257; doi:10.3390/ijerph18136791)
Supplement: Supplementary file 1 [file ijerph-18-06791-s001.zip › ijerph-1264024-supplementary.pdf]

Table S1. The Kihon Checklist in English

No.	Questions	Answer	
1	Do you go out by bus or train by yourself?	□0. YES	□1. NO
2	Do you go shopping to buy daily necessities by yourself?	□0. YES	□1. NO
3	Do you manage your own deposits and savings at the bank?	□0. YES	□1. NO
4	Do you sometimes visit your friends?	□0. YES	□1. NO
5	Do you turn to your family or friends for advice?	□0. YES	□1. NO
6	Do you normally climb stairs without using handrail or wall for support?	□0. YES	□1. NO
7	Do you normally stand up from a chair without any aids?	□0. YES	□1. NO
8	Do you normally walk continuously for 15 minutes?	□0. YES	□1. NO
9	Have you experienced a fall in the past year?	□1. YES	□0. NO
10	Do you have a fear of falling while walking?	□1. YES	□0. NO
11	Have you lost 2 kg or more in the past 6 months?	□1. YES	□0. NO
12	Height: cm, weight: kg, BMI: kg/m^2 If BMI is less than 18.5, this item is scored.	□1. YES	□0. NO
13	Do you have any difficulties eating tough foods compared to 6 months ago?	□1. YES	□0. NO
14	Have you choked on your tea or soup recently?	□1. YES	□0. NO
15	Do you often experience having a dry mouth?	□1. YES	□0. NO
16	Do you go out at least once a week?	□0. YES	□1. NO
17	Do you go out less frequently compared to last year?	□1. YES	□0. NO
18	Do your family or your friends point out your memory loss? e.g."You ask the same question over and over again."	□1. YES	□0. NO
19	Do you make a call by looking up phone numbers?	□0. YES	□1. NO
20	Do you find yourself not knowing today's date?	□1. YES	□0. NO
21	In the last 2 weeks have you felt a lack of fulfilment in your daily life?	□1. YES	□0. NO
22	In the last 2 weeks have you felt a lack of joy when doing the things you used to enjoy?	□1. YES	□0. NO
23	In the last 2 weeks have you felt difficulty in doing what you could do easily before?	□1. YES	□0. NO
24	In the last 2 weeks have you felt helpless?	□1. YES	□0. NO
25	In the last 2 weeks have you felt tired without a reason?	□1. YES	□0. NO

Working Group on Frailty in Japanese Geriatrics Society. BMI, body mass index.

Arai H, Satake S. English translation of the Kihon Checklist. Geriatr Gerontol Int 2015;15(4):518-9. doi: 10.1111/ggi.12397 [published Online First: 2015/04/02]
